# Connexin43 Is Required for the Effective Activation of Spleen Cells and Immunoglobulin Production

**DOI:** 10.3390/ijms20225789

**Published:** 2019-11-18

**Authors:** Yanru Huang, Zhimin Mao, Xiling Zhang, Xiawen Yang, Norifumi Sawada, Masayuki Takeda, Jian Yao

**Affiliations:** 1 Division of Molecular Signaling, Department of the Advanced Biomedical Research, Interdisciplinary Graduate School of Medicine, University of Yamanashi, Chuo 409-3898, Japan; huangyanru90s@outlook.com (Y.H.); mao1100@outlook.com (Z.M.); xilingzhang@126.com (X.Z.); g18dim07@yamanashi.ac.jp (X.Y.); 2 Department of Urology, Interdisciplinary Graduate School of Medicine, University of Yamanashi, Chuo 409-3898, Japan; nsawada@yamanashi.ac.jp (N.S.); matakeda@yamanashi.ac.jp (M.T.)

**Keywords:** connexin43, immunoglobulin, spleen cells, B cells, oxidative stress

## Abstract

Gap junctions (Gjs), formed by specific protein termed connexins (Cxs), regulate many important cellular processes in cellular immunity. However, little is known about their effects on humoral immunity. Here we tested whether and how Gj protein connexin43 (Cx43) affected antibody production in spleen cells. Detection of IgG in mouse tissues and serum revealed that wild-type (Cx43^+/+^) mouse had a significantly higher level of IgG than Cx43 heterozygous (Cx43^+/−^) mouse. Consistently, spleen cells from Cx43^+/+^ mouse produced more IgG under both basal and lipopolysaccharide (LPS)-stimulated conditions. Further analysis showed that LPS induced a more dramatic activation of ERK and cell proliferation in Cx43^+/+^ spleen cells, which was associated with a higher pro-oxidative state, as indicated by the increased NADPH oxidase 2 (NOX2), TXNIP, p38 activation and protein carbonylation. In support of a role of the oxidative state in the control of lymphocyte activation, exposure of spleen cells to exogenous superoxide induced Cx43 expression, p38 activation and IgG production. On the contrary, inhibition of NOX attenuated the effects of LPS. Collectively, our study characterized Cx43 as a novel molecule involved in the control of spleen cell activation and IgG production. Targeting Cx43 could be developed to treat certain antibody-related immune diseases.

## 1. Introduction

Gap junctions (Gjs) are intercellular channels that directly link the cytoplasm of the adjacent cells. They are formed by a specific family of proteins termed connexin (Cx). Six Cx molecules form a hemichannel. Docking of two hemichannels in apposed membranes of two neighboring cells forms an intact Gj channel. Up to now, more than 20 different isoforms of Cxs have been reported. Among them, connexin43 (Cx43) has been the focus of many investigations because it is predominantly and ubiquitously expressed in many cell types. Intercellular communication mediated by Gjs provides a pathway for the intercellular exchange of signaling molecules, which is fundamentally essential for the maintenance of homeostasis in tissues and organs and has been shown to regulate a wide range of cellular processes and functions [1,2].

In the immune system, there exist extensive interactions among immunocompetent cells and the surrounding environment, which are mediated by paracrine cytokines, adhesion molecules, matrixes and direct cell-to-cell communication. Although the roles of adhesion molecules and paracrine cytokines in the immune system have been extensively documented, our knowledge about Gjs in the immune system is still limited. Several studies indicate that Cx43 is expressed in a variety of immunocompetent cells and participates in the regulation of immune cell functions, such as migration, phagocytosis, antigen presentation, T/B cell reactivity and cytokine production [3,4,5,6,7,8,9,10,11].

Most of the studies regarding Cx proteins and channels in the regulation of immune processes and events have been focused on cellular immunity. Researches about the roles of Cxs in humoral immunity remain scarce. Humoral immunity refers to mechanisms of the adaptive immune defenses mediated by antibodies secreted by B lymphocytes. Several lines of evidence prompted us to speculate that Cx43 may be critically involved in the regulation of humoral immunity. First, Cx43 is known to be expressed in spleen B lymphocytes and contribute to the regulation of B cell adhesion, spreading and activation [10]. Second, Cx43 modulates intracellular redox status through multiple mechanisms. It regulates two important redox protein NADPH oxidase (NOX) and thioredoxin-interacting protein (TXNIP) in several different cell types [4,12,13,14,15]. Interestingly, a pivotal role of NOX and TXNIP in the control of reactive oxygen species (ROS) and B cell activation has been reported [16,17,18]. Third, Cx43 is also implicated in the regulation of many cellular events that are directly or indirectly related to B cell activation in vivo. These events include nuclear factor kappa-light-chain-enhancer of activated B cells (NF-κB) activation, inflammasome formation, dendritic cell (DC) and T cell function, cytokine production and the process of antigen presentation [3,4,5,6,7,8,9,10,11,19]. These considerations strongly support the idea that Cx43 may be critically involved in the control of B cell activation and immunoglobulin (Ig) production. The purpose of this study was to address this hypothesis.

Here, we present our data showing that Cx43 regulates IgG production by spleen cells through mechanisms involving its modulation on the intracellular redox status. Our study provides additional evidence supporting the involvement of Cx43 in the regulation of immune responses and suggests that Cx43 could be targeted to treat certain antibody-associated diseases.

## 2. Results

### 2.1. Cx43^+/+^ Has a Higher Level of Serum IgG Than Cx43^+/−^ Mouse

**Figure 1:**
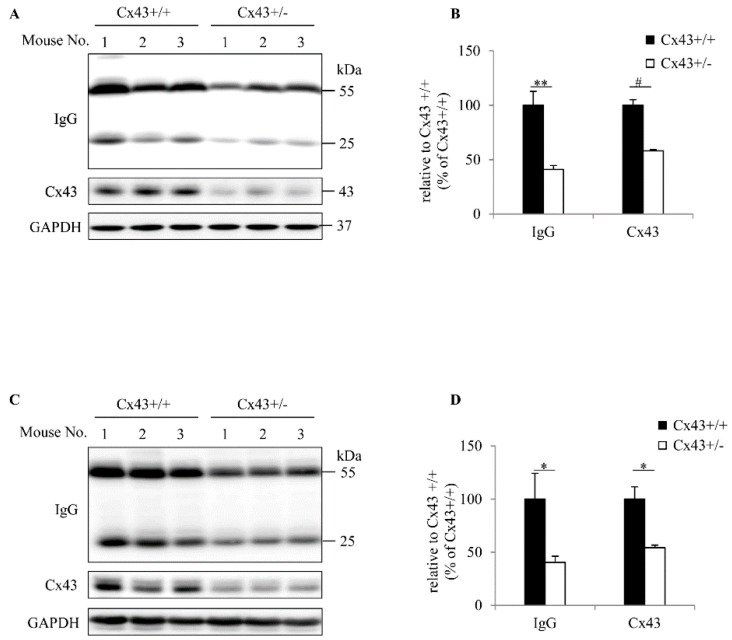
Immunoglobulin (IgG) levels in the tissue samples of connexin (Cx)43^+/+^ and Cx43^+/−^ mice. Protein lysates extracted from the normal tissues of Cx43^+/+^ and Cx43^+/−^ mice were subjected to Western blot analysis for IgG and Cx43. GAPDH was used as an internal control. Level of IgG and Cx43 in liver and kidney was shown in (**A**) and (**C**), respectively. The densitometric quantitation of IgG and Cx43 bands in (**A**) and (**C**) are shown in (**B**) and (**D**). Data shown are mean ± SE (*n* = 10 for IgG; *n* = 3 for Cx43). * *p* < 0.05, ** *p* < 0.01, # *p* < 0.001.

During our Western blot analysis of the denatured tissue samples from Cx43^+/+^ and Cx43^+/−^ mice with anti-mouse IgG antibodies, we noticed two nonspecific bands at the molecular weight of 55 and 25 kDa, which was consistently different between Cx43^+/+^ and Cx43^+/−^ mice. The location of the bands corresponds to IgG heavy and light chains. This observation prompted us to investigate whether there was a difference in IgG between Cx43^+/+^ and Cx43^+/−^ mice. We, therefore, conducted a more detailed analysis. Figure 1A–D show there existed a visible difference in IgG among the tissue samples extracted from the normal livers and kidneys of Cx43^+/+^ and Cx43^+/−^ mice. The level of tissue IgG was significantly higher in Cx43^+/+^ mice than Cx43^+/−^ mice.

**Figure 2:**
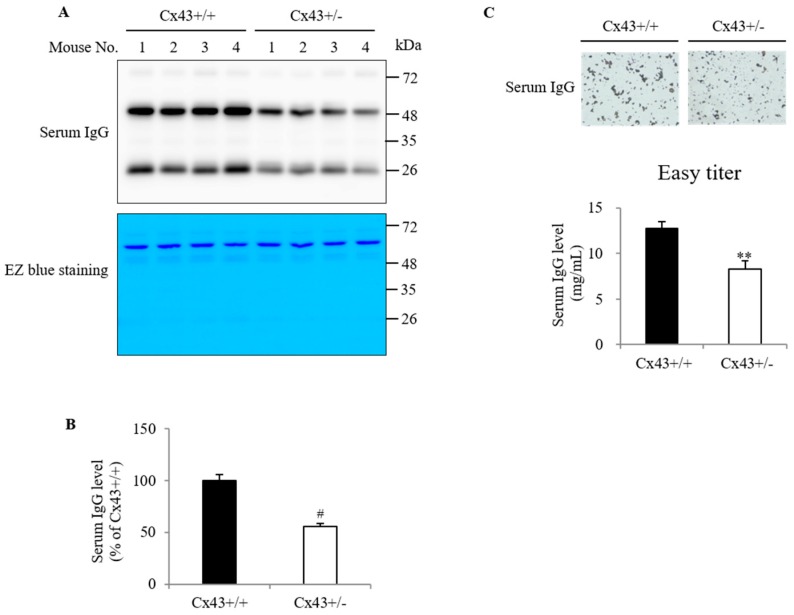
Serum IgG levels in Cx43^+/+^ and Cx43^+/−^ mice. Serum IgG levels in Cx43^+/+^ and Cx43^+/−^ mice were determined using Western blot analysis (**A**,**B**) and Easy-Titer assay (**C**). The membrane used for Western blot analysis was also stained with EZ blue for confirmation of the equal loading of the samples (**A**: lower panel). Note the same intensity of the band at MW around 66 kDa between Cx43^+/+^ and Cx43^+/−^ mice. The densitometric quantitation of IgG bands in (**A**) is shown in (**B**). Data shown are mean ± SE (*n* = 10). # *p* < 0.001. (**C**) Determination of serum IgG concentration using Easy-Titer assay. Serum samples were allowed to react with anti-mouse IgG-coated microbeads, as described in the section of Materials and Methods. The agglutination of the microbeads was photographed (**C**: upper part; magnification 200×). Note the obvious difference in the size of the aggregates after reaction with Cx43^+/+^ and Cx43^+/−^ mouse serum. The concentration of serum IgG calculated from the standard curve, generated from monoclonal antibody 1-22-3, is shown in (**C**) (lower part). Data shown are mean ± SE (*n* = 4). ** *p* < 0.01.

The different amount of IgG in the normal tissue homogenates could come from the residual blood serum left in the tissue vasculature; we, therefore, detected the serum IgG using Western blot analysis and Easy-Titer assay. Figure 2A shows that the IgG bands at the molecular weight (MW) of 55 and 25 kDa in Western blot were stronger in Cx43^+/+^ serum than Cx43^+/−^ serum. Staining of the proteins in the membrane with EZ blue revealed that there was no difference in serum albumin, the predominant band at the location of 66 kDa, suggesting that the difference in serum IgG was relatively specific and not caused by the discrepancy in the loaded protein.

Consistent with the Western blot result, direct measurement of serum IgG with Easy-Titer assay also showed a significant difference in serum IgG between Cx43^+/+^ and Cx43^+/−^ mice. This assay is based on the principle that the reaction of sample IgG with the antibody-sensitized polystyrene beads results in the formation of aggregates, which causes a reduction in the light absorption at 405 nm in a way proportional to the IgG concentration. Figure 2C shows that the size of the aggregates formed by Cx43^+/+^ serum was larger than that by Cx43^+/−^ serum, suggesting a higher level of serum IgG in Cx43^+/+^.

### 2.2. Cx43^+/+^ Spleen Cells Produce More IgG Than Cx43^+/−^ Cells

**Figure 3:**
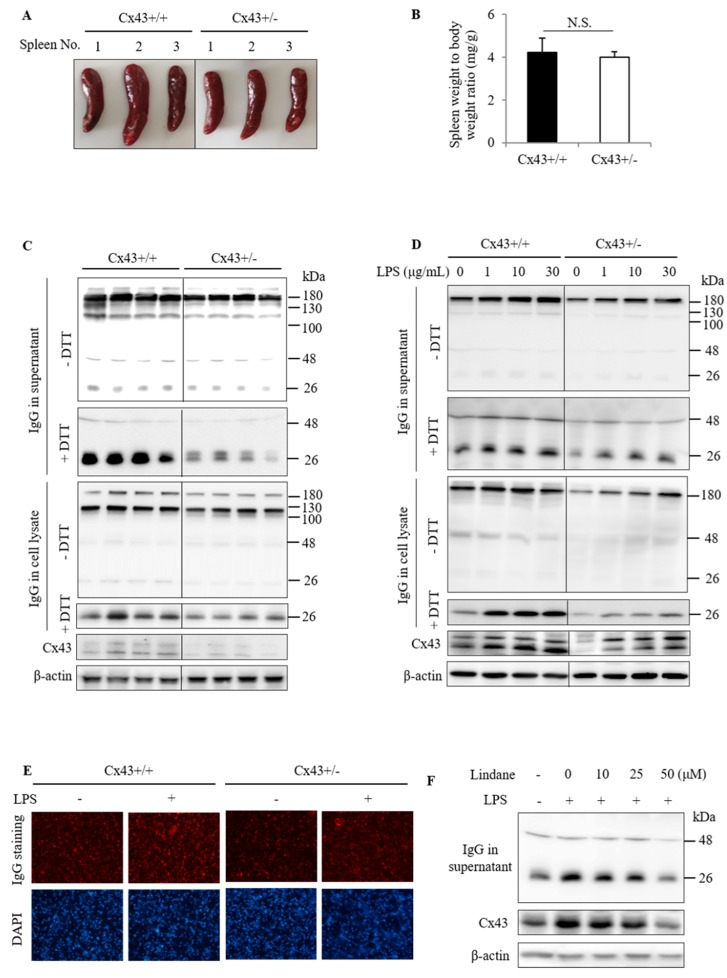
Influence of Cx43 on spleen size and spleen cell production of IgG. (**A**,**B**) Spleen size and weight in Cx43^+/+^ and Cx43^+/−^ mice. Spleen obtained from Cx43^+/+^ and Cx43^+/−^ mice was photographed (**A**). The ratio of the spleen to bodyweight was shown in (**B**). Data are mean ± SE (*n* = 10). (**C**,**D**) IgG production by spleen cells from Cx43^+/+^ and Cx43^+/−^ mice under basal and lipopolysaccharide (LPS)-stimulated condition. The spleen cells isolated from Cx43^+/+^ and Cx43^+/−^ mice were cultured either for 2 days at basal condition (**C**) or in the presence of the indicated concentrations of LPS for 24 h (**D**). Supernatants and cell lysates were collected and analyzed for IgG by Western blot under native and denatured conditions. Protein level of Cx43 in the lysates was also detected. β-actin was used as an internal control. (**E**) Immunofluorescence staining of IgG in spleen cells from Cx43^+/+^ and Cx43^+/−^ mice. The spleen cells were either left untreated or incubated with 20 μg/mL LPS for 16 h, and then subjected to immunofluorescence staining of IgG (red) and nuclear staining with DAPI (blue; magnification 200×). Note the different fluorescent intensity in Cx43^+/+^ and Cx43^+/−^ spleen cells. (**F**) Effect of Cx43 inhibitor on LPS-induced IgG secretion. The spleen cells were stimulated with 20 μg/mL LPS in the presence or absence of the indicated concentrations of Lindane for 24 h. The collected supernatant and cell lysate were subjected to Western blot analysis for IgG and Cx43.

The difference in serum IgG led us to explore the underlying mechanisms. Given that spleen plasma cells are one of the major sources of serum IgG, we examined the possible difference between Cx43^+/+^ and Cx43^+/−^ spleen. Figure 3A shows that the spleen of Cx43^+/+^ mice appeared to be slightly bigger than Cx43^+/−^ mice. However, statistical analysis showed that there was no significant difference between Cx43^+/+^ and Cx43^+/−^ mice in the ratio of spleen to body weight (Figure 3B).

We then proceeded to examine IgG production by cultured spleen cells under both basal and lipopolysaccharide (LPS)-stimulated conditions [20,21,22]. Figure 3C shows that spleen cells isolated from Cx43^+/+^ mice produced more IgG than Cx43^+/−^ mice. LPS stimulated IgG production and secretion in both Cx43^+/+^ and Cx43^+/−^ spleen cells in a concentration-dependent manner. The elevation was also more pronounced in Cx43^+/+^ cells than Cx43^+/−^ cells. Intriguingly, LPS also elevated Cx43 (Figure 3D).

Immunofluorescent staining of the cultured spleen cells with Rhodamine-conjugated anti-mouse IgG antibody showed that most of the cells were positively stained, indicating that they were B lymphocytes. Consistent with the results of Western blot analysis, Cx43^+/+^ spleen cells displayed stronger staining of IgG than Cx43^+/−^ cells under basal and LPS-stimulated conditions (Figure 3E).

To further confirm the role of Cx43 in the control of IgG production, we used Gj inhibitor lindane. As previously reported in other cell types [23], lindane also suppressed Cx43 levels in spleen cells. In the presence of lindane, LPS-induced elevation in IgG was inhibited (Figure 3F). Collectively, these observations indicate that Cx43 regulates IgG production by isolated spleen cells.

### 2.3. Cx43^+/+^ Spleen Cells Display a Stronger Response to LPS Than Cx43^+/−^ Cells in Cell Proliferation and Redox Signaling

**Figure 4:**
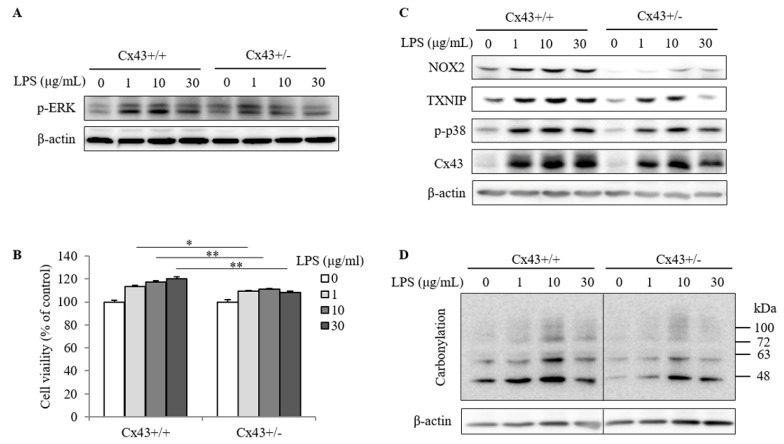
Responses of Cx43^+/+^ and Cx43^+/−^ spleen cells to LPS-induced changes in proliferation and redox status. (**A**,**B**) Different cell responses to LPS-induced ERK activation and cell proliferation. Spleen cells isolated from Cx43^+/+^ and Cx43^+/−^ mice were stimulated with the indicated concentrations of LPS for 24 h. Cell lysates were collected and detected for p-ERK using Western blot analysis (**A**). Cell proliferation after the stimulation was determined using WST assay (**B**). Data are expressed as the percentage of formazan formation against the untreated control (mean ± SE, *n* = 4). * *p* < 0.05, ** *p* < 0.01. (**C**) The response of Cx43^+/+^ and Cx43^+/−^ spleen cells to LPS-induced changes in intracellular oxidative status. Spleen cells were treated the same as above. The cellular proteins were harvested and subjected to Western blot analysis for NOX2, TXNIP, p-p38 and Cx43 (**C**), as well as protein carbonylation (**D**). Data in (**C**) were representative of three separate experiments with similar results.

LPS-induced lymphocyte activation is associated with an increased lymphocyte proliferation [22]. We, therefore, tested the possible difference between Cx43^+/+^ and Cx43^+/−^ spleen cells. Figure 4A and B show that stimulation of spleen cells with LPS caused an ERK activation (a signaling molecule that is closely related to cell mitogenesis) and cell proliferation, which was significantly more vigorous in Cx43^+/+^ than Cx43^+/−^ cells.

Given that ROS plays a central role in the activation of immunocompetent cells [4,16,17,24] and that Cx43 regulates intracellular redox state through multiple mechanisms [4,12,13,14,15]; we, therefore, examined the possible difference in redox status. Figure 4C and D show that LPS-induced activation of spleen cells was associated with an increased NOX2 and TXNIP, two pivotal molecules involved in the control of intracellular ROS and redox signaling. Consistently, there was also an increase in p38 activation and protein carbonylation, suggesting that LPS induced spleen cells into the pro-oxidative state. Intriguingly, Cx43^+/+^ spleen cells displayed more dramatic changes in these oxidative parameters, as compared to Cx43^+/−^ cells.

### 2.4. ROS Contributes to Spleen Cell Activation and IgG Production

**Figure 5:**
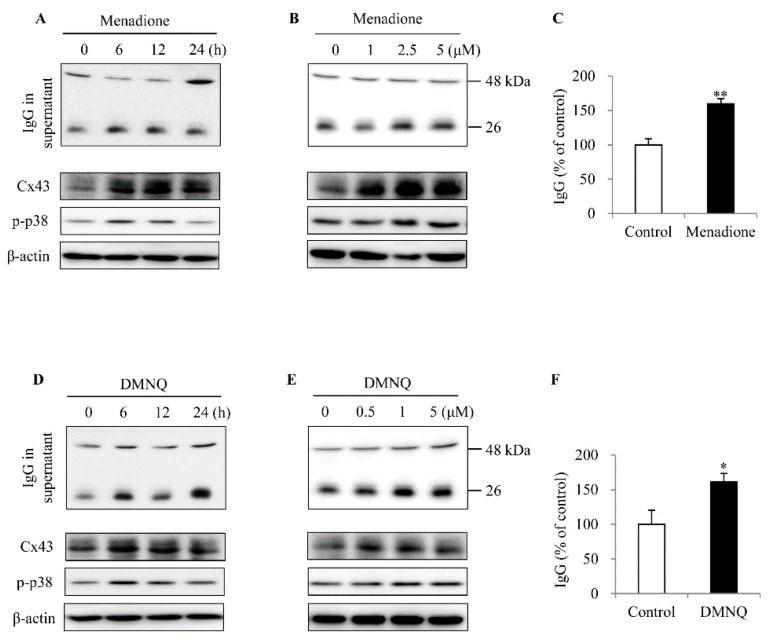
Effect of superoxide donors on IgG production, Cx43 expression and p38 activation in cultured spleen cells. (**A**,**B**,**D**,**E**) Induction of IgG, Cx43 and p38 activation by superoxide donors. Spleen cells isolated from Cx43^+/+^ mice were stimulated with 2.5 μM menadione or 0.5 μM DMNQ for the indicated time or exposed to the indicated concentrations of the reagents for 24 h. Supernatants and cell lysates were harvested and subjected to Western blot analysis for IgG, Cx43 and p-p38. The quantitation of IgG at 24 h point after treatment with 2.5 μM menadione and 0.5 M DMNQ was shown in (**C**) and (**F**), respectively. Data shown are mean ± SE (*n* = 3). * *p* < 0.05, ** *p* < 0.01.

To determine the role of the pro-oxidative state in IgG production, we exposed spleen cells to two different exogenous superoxide donors, menadione and 2,3-dimethoxy-1,4-naphthoquinone (DMNQ), and found that these chemicals stimulated IgG secretion, together with an induction of p38 activation and Cx43 protein level in a way similar to LPS (Figure 5A–F).

**Figure 6:**
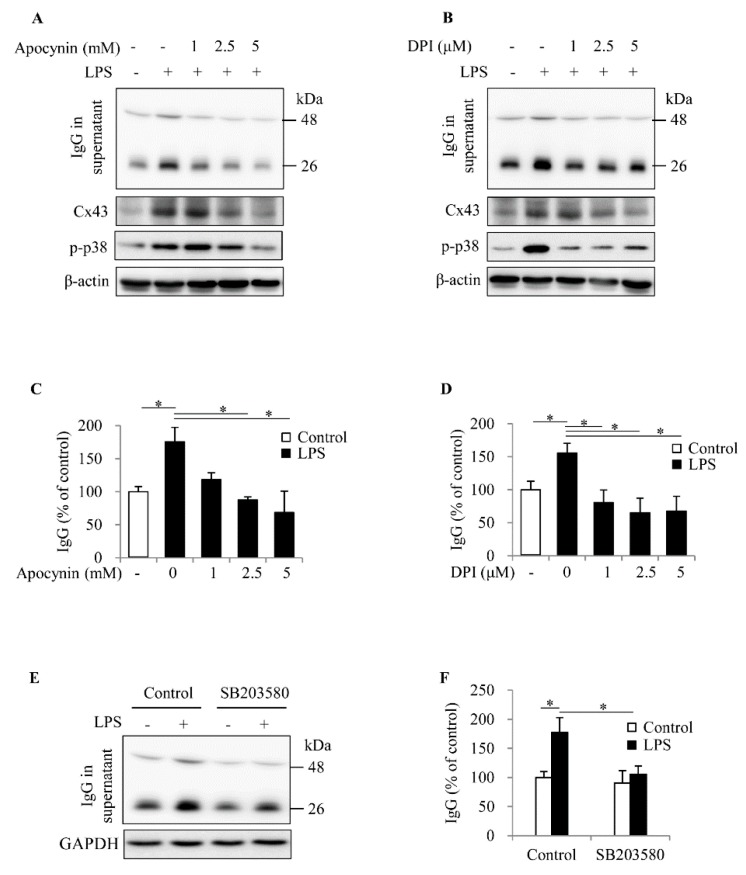
Effects of inhibition of NADPH oxidase (NOX) and p38 on LPS-induced production of IgG. (**A**) The isolated spleen cells were stimulated with 20 μg/mL LPS in the presence or absence of the indicated concentrations of NOX inhibitors, APO and DPI, for 24 h. Supernatants and cellular proteins were harvested and assayed for IgG, Cx43 and p-p38 using Western blot analysis. The densitometric quantitation of IgG in (**A**) and (**B**) are shown in (**C**) and (**D**). Data shown are mean ± SE (*n* = 3). * *p* < 0.05. (**E**,**F**) Effects of p38 inhibitor on LPS-initiated IgG secretion. The isolated spleen cells were treated with 20 μg/mL LPS in the presence or absence of 10 μM SB203580 for 24 h. Supernatants were collected and assayed for IgG. Cellular protein was assayed and used as an internal control. The densitometric quantitation of IgG in (**E**) is shown in (**F**). Data shown are mean ± SE (*n* = 3). * *p* < 0.05.

To further establish the role of ROS in spleen cell activation, we inhibited NOX, which is known to play a vital role in ROS generation and B cell activation [16,17]. Figure 6A–D show that inhibition of NOX with apocynin and DPI almost completely abolished the effects of LPS on IgG production, Cx43 expression and p38 activation.

P38 has been described to mediate B cell activation, proliferation, antibody production and secretion [25,26,27,28]. To confirm an involvement of oxidative kinase p38 in spleen cell activation, we treated the spleen cells with p38 inhibitor SB203580 and found the IgG secretion induced by LPS was obviously suppressed (Figure 6E,F). These observations indicate that NOX-mediated production of superoxide and p38 contribute to LPS-induced spleen cell activation and IgG production.

## 3. Discussion

In this study, we characterized Cx43 as an unrecognized molecule involved in the control of spleen cell activation and IgG production, possibly through modulation of the intracellular redox status. Our study, thus, provides novel evidence supporting a vital role of Cx43 in the regulation of immune functions.

Cx43 is expressed in a variety of immuno-potent cells and participates in the regulation of many immune reactions [3,4,5,6,7,8,9,10,11]. It influences neutrophil recruitment, macrophage activation, DC maturation, B cell and T cell development. It stimulates the production of inflammatory mediators and the expression of adhesion molecules. Many important cellular events involved in the regulation of immune cell functions, such as NF-κB activation and inflammasome formation are also influenced by Cx43 [4,29]. Inhibition or downregulation of Cx43 alleviates the local inflammatory reactions and accelerates the recovery process [30,31,32]. Currently, the studies regarding Cx43 on immune functions have been focused mainly on its actions on cellular immunity. In this study, we found that Cx43 also participated in the regulation of humoral immunity.

IgG, produced by B lymphocytes, is one of the major effector molecules in humoral immunity. In this study, we observed, for the first time, that there existed a significant difference in serum IgG between Cx43^+/+^ and Cx43^+/−^ mice. Furthermore, we demonstrated that the difference also existed in cultured spleen cells. As the largest secondary lymphoid organ in the body, the spleen is known to be involved in B cell maturation and IgG production in vivo. Our observation thus suggested that the difference in serum IgG was most likely the result of Cx43 on antibody-producing B lymphocytes. In support of this notion, the expression of Cx43 in mouse B lymphocytes and its participation in B lymphocyte activation have been reported [3,10]. Cx43 facilitated B cell adhesion, spreading and migration initiated by B cell receptor (BCR) activation. In addition, blockade of Cx43 channels markedly reduced the secretion of immunoglobulin G (IgG), IgM, and IgA in mixed cultures of purified human T and B lymphocytes [33,34]. In this study, we also demonstrated that Cx43 contributed to LPS induced spleen cell activation, as evidenced by the increased ERK phosphorylation and cell proliferation. Cx43 is required for the effective activation of B lymphocytes and IgG production.

How did Cx43 regulate the activation and IgG production of spleen cells? This question remains to be answered. Lymphocyte activation is associated with activation of many signaling molecules, which are known to be controlled by the concerted actions of kinases and phosphatases [35]. Recently, ROS has been identified as one of the major regulators of kinases and phosphatases. The activation of redox signaling by ROS has been established as the central mechanisms underlying the activation of multiple immunocompetent cells, including macrophages and B lymphocytes [4,35]. Activation of BCR is associated with an increased ROS generation [16,17]. Inhibition of ROS or NOX2 (the major isoform of superoxide-generating NOX in B lymphocytes) attenuates B cell activation. In line with these previous reports, we also noticed that a higher prooxidative state induced by LPS contributed to spleen cell activation. This conclusion is supported by the observation that LPS caused elevation in NOX2, TXNIP, p-p38 and direct addition of the exogenous free radicals into cultured spleen cells, mimicking the effects of LPS on IgG and Cx43, whereas inhibition of NOX abolished the effect of LPS. In this context, it is reasonable to assume that the effect of Cx43 on spleen cell activation and IgG production was through its modulation on intracellular redox status.

The question naturally occurred as to how Cx43 regulated intracellular redox status. Studies from our group and others showed that Cx43 modulated intracellular state via both communication-dependent and -independent mechanisms. Cx43 channel-mediated intercellular communication amplified Ca^2+^ signaling, a key molecule implicated in the induction of ROS [36]. It also transmitted and propagated ROS among multiple cells [37]. In addition, Gj hemichannels could also be activated under oxidative and inflammatory situations. The loss of ATP and the major antioxidant GSH and NOX could further exaggerate the intracellular oxidative condition [15,38,39]. Moreover, Cx43 also worked through communication-independent mechanisms, possibly through direct interaction with important structural and functional proteins [40]. We have reported that Cx43 regulated TXNIP and NOX4 in renal cells via a communication-independent way [4,12,14]. Collectively, Cx43 may regulate the intracellular redox status of spleen cells through multiple mechanisms.

Currently, the mechanisms involved in the control of spleen cell activation by Cx43 is unclear and remains to be clarified in the future. However, we speculated that it could be closely related to its regulatory actions on NOX. In our previous studies, we have demonstrated that Cx43 regulated NOX2/NOX4 in macrophages and renal resident cells [4,12,13,14,15]. Furthermore, we also revealed the existence of a reciprocal regulation between Cx43, NOX, and p38 activation in several different cell types [4,14,15]. It appeared that the same regulatory loop also existed in spleen cells and contributed to the observed effects of Cx43 on spleen cell activation. This conclusion was evidenced by the fact that Cx43-deficient spleen cells exhibited a blunted oxidative response to LPS, as revealed by the lower level of NOX2 and p-p38, and that the inhibition of NOX caused a reduction in Cx43 and p38 activation. Given that NOX2 is one of the major isoforms of NOX contributing to ROS production and B lymphocyte activation, the reduced level of NOX2 in Cx43^+/−^ cells, as well as Gj inhibitor lindane-treated cells (data not shown), suggested that the observed effect of Cx43 on IgG production could be ascribed to its action on NOX2 in spleen cells.

Of note, in this study, we also demonstrated a clear difference in the protein level of TXNIP between Cx43^+/+^ and Cx43^+/−^ spleen cells under both basal and LPS-stimulated conditions. Intriguingly, the difference also existed in vivo (Supplementary Figure S1). As an inhibitor of Trx, TXNIP (thioredoxin interacting protein), participates in the control of stress responses, redox regulation, cellular proliferation and metabolism [12,13]. It is also involved in the formation of germinal centers in peripheral lymphoid organs. TXNIP (−/−) mice exhibited a reduction in antibody production and plasma cell numbers [18]. Recently, regulation of NOX by TXNIP was reported [41,42]. It is conceivable that Cx43-mediated regulation of TXNIP could be an important mechanism behind its regulatory effects on spleen cell activation and IgG production.

Several studies documented a critical involvement of p38 activation in B cell activation, proliferation, antibody production and secretion [25,26,27,28]. As a downstream signaling molecule of TXNIP/thioredoxin (Trx)/Apoptosis signal-regulating kinase (ASK)1 signaling pathway, we have documented that p38 was under the control of Cx43 in several different cell types [4,12,14]. In this study, p38 activation between Cx43^+/+^ and Cx43^+/−^ spleen cells was also different. Regulation of oxidative stress and oxidative sensitive p38 signaling pathway could be a common mechanism by which Cx43 exerts its biological actions on a variety of cells.

It is also worth noting that Cx43 is expressed in many immunocompetent cells and participates in the regulation of many molecular events, such as activation of antigen-presenting DC and T cells, induction of cytokine production, promotion of NF-κB and inflammasome activation [7,43]. All these molecular events have been shown to affect B cell activation and IgG production in one way or another. It is conceivable that the observed effects of Cx43 on IgG production, especially in in vivo situation, could be a result of the combined actions of Cx43 on many different cell types and multiple target molecules.

In conclusion, our study characterized Cx43 as a novel molecule involved in the control of spleen cell activation and IgG production. Given that antibody is one of the major effector molecules in humoral immunity and is implicated in many immune-related diseases, targeting Cx43 could be developed to treat certain antibody-related diseases.

## 4. Materials and Methods

### 4.1. Materials

OxyBlot™ protein oxidation detection kit was purchased from Merck Millipore (EMD Millipore, Billerica, MA, USA). WST reagent was from Dojindo (Kumamoto, Japan). SB203580 was obtained from Calbiochem (San Diego, CA, USA). Rhodamine-conjugated donkey anti-mouse IgG was from Santa Cruz Biotechnology (Dallas, TX, USA). Antibodies against β-tubulin, GAPDH, phospho-p38, p-ERK, TXNIP, as well as horseradish peroxidase-conjugated anti-rabbit or anti-mouse IgG were obtained from Cell Signaling Technology (Danvers, MA, USA). NOX2 was bought from Bioss (Tokyo, Japan). DMNQ was from Nacalao Tesque (Kyoto, Japan). EZ blue gel staining reagent, LPS, Lindane, apocynin, diphenyleneiodoniumchloride (DPI), Menadione, anti-Cx43, anti-β-actin, and all other chemicals were from Sigma (Tokyo, Japan).

### 4.2. Animals

Cx43 wild-type (WT, Cx43^+/+^) and heterozygous Cx43 (Cx43^+/−^) mice at the bodyweight between 20 to 25 g were from the offspring of Cx43^+/+^ mice mated with heterozygous Cx43 mice (Cx43^+/−^; B6; 129-Gja1<tm1Kdr>/J; Jackson Laboratories, Bar Harbor, ME, USA). The mice were housed in the Animal Center of the University of Yamanashi and were allowed to get access to food and water freely in an air-conditioned room with a 12-h light/dark cycle. The genotypes of all mice were performed by polymerase chain reaction (PCR), based on the protocol provided by Jackson Laboratories. All animal experiments were approved by the animal experiment committee of our university (A26-23, approval on 5 March 2015) and performed following the relevant guidelines and regulations.

### 4.3. Cells

Spleen cell suspension was made by dissociation of spleen tissue with forceps. Single splenocyte was obtained by gently pipetting cell suspension. Afterward, red blood cells (RBC) were removed by adding RBC lysis buffer (Sigma, Saint Louis, MO, USA). Splenocytes were cultured in a plate at the density of 5–6 × 10^6^ in RPMI 1640 medium containing 10% fetal bovine serum (FBS; Sigma-Aldrich, Carlsbad, CA, USA) and 1% penicillin/streptomycin/antibiotic antimycotic solution (ABAM; Sigma-Aldrich, Carlsbad, CA, USA).

### 4.4. Western Blot Analysis

The sample preparation and Western blot analysis were performed as we previously reported [4]. Briefly, liver and kidney tissues were homogenized in urea buffer (8 mol/L urea, 1 mmol/L dithiothreitol, 1 mmol/L ethylenediaminetetraacetic acid, 50 mmol/L Tris-HCl at pH 8.0) on ice. Lysates were sonicated and centrifuged at 12,000 rpm for 30 min at 4 °C. Supernatants were collected and assayed for protein concentration with Pierce Micro BCA Protein Assay Kit (Thermo Fisher Scientific, Waltham, MA, USA). For cells, the cellular protein was extracted with 1× SDS sample buffer. The same amount of protein was loaded and separated by SDS-polyacrylamide gels and electrotransferred onto polyvinylidene difluoride membranes, which were followed by blocking the membranes with 5% non-fat dry milk in PBS and incubation with primary antibody overnight at 4 °C. After washing, the membranes were probed with horseradish peroxidase-conjugated anti-rabbit or anti-mouse IgG, and the bands were developed using Chemi-Lumi One L (Nacalai Tesque, Kyoto, Japan) and captured with a Fujifilm luminescent image LAS-1000 analyzer (Fujifilm, Tokyo, Japan). The results were quantified using Image J software (National Institutes of Health, Bethesda, MD, USA). β-actin or GAPDH was used as internal loading control.

### 4.5. Easy-Titer IgG Assay

Easy-Titer Mouse IgG Assay Kit (Pierce, Waltham, MA, USA) was used for determination of serum IgG concentration. The assay was performed as we have previously reported [44]. Briefly, the anti-IgG-sensitized beads were allowed to react with the same volume of the diluted serum samples or standard IgG in a 96-well plate. After vigorous mixing on a plate mixer, blocking reagent was added. The OD at 405 nm was measured using a UV/VIS spectrometer. The concentration of IgG was calculated based on the standard curve made from standard mouse IgG. The formation of microagglutination of the microbeads was also photographed using a CCD camera attached to an Olympus BX50 microscope (Olympus, Tokyo, Japan).

### 4.6. Polymerase Chain Reaction

Mouse genotyping was performed with PCR using a protocol provided by Jackson Laboratories (Bar Harbor, ME, USA), as we have previously reported [4]. Briefly, DNA was extracted from ear punch tissue and was analyzed by PCR. The products of PCR were loaded on an agarose gel. The expected MW of PCR products was 320 and 600 bp for heterozygote mouse, and 600 bp for Wild-type mouse.

### 4.7. Assessment of Cell Proliferation with WST Reagent

The isolated spleen cells of Cx43^+/+^ and Cx43^+/−^ mice were seeded into 96-well culture plates and exposed to the indicated concentrations of LPS for 24 h. WST reagent was added into each well 2–3 h before measurement of OD with a spectrometer at the wavelength of 450 nm. Cell proliferation was expressed as a percentage of control cells.

### 4.8. Immunofluorescent Staining of IgG in Spleen Cells

Spleen cells stimulated with or without LPS were incubated with Rhodamine-conjugated donkey anti-mouse IgG (1:200) for 2 h, followed by DAPI staining for an additional 10 min. After washing with PBS, the cells were observed under the fluorescent microscope (BX50; Olympus, Tokyo, Japan) and the fluorescent signal was captured with a CCD camera attached to the microscope.

### 4.9. Statistical Analysis

Values are expressed as mean ± SE. Comparison of two groups was made by Student’s *t*-test. The analysis was performed using Microsoft Excel (Microsoft, Redmond, WA, USA). *p* < 0.05 was considered statistically significant.

## 5. Conclusions

In conclusion, our study characterized Cx43 as a novel molecule involved in the control of spleen cell activation and IgG production. Given that antibody is one of the major effector molecules in humoral immunity and is implicated in many immune-related diseases, targeting Cx43 could be developed to treat certain antibody-related diseases.

## Supplementary Materials

Supplementary materials can be found at https://www.mdpi.com/1422-0067/20/22/5789/s1.

## Abbreviations

ASK	Apoptosis signal-regulating kinase
Cx	Connexin
Cx43	Connexin43
DMNQ	2,3-Dimethoxy-1,4-naphthoquinone
DC	Dendritic cell
GJ	Gap junction
Ig	Immunoglobulin
IgG	Immunoglobulin G
LPS	Lipopolysaccharide
MW	Molecular weight
NADPH oxidase	NOX
NF-κB	Nuclear factor kappa-light-chain-enhancer of activated B cells
ROS	Reactive oxygen species
Trx	Thioredoxin
TXNIP	Thioredoxin-interacting protein
