# Supplementary material for: Connexin43 Is Required for the Effective Activation of Spleen Cells and Immunoglobulin Production
Source: Int J Mol Sci. 2019 Nov 18;20(22):5789. doi: 10.3390/ijms20225789 (PMC6888161; doi:10.3390/ijms20225789)
Supplement: Supplementary file 1 [file ijms-20-05789-s001.pdf]

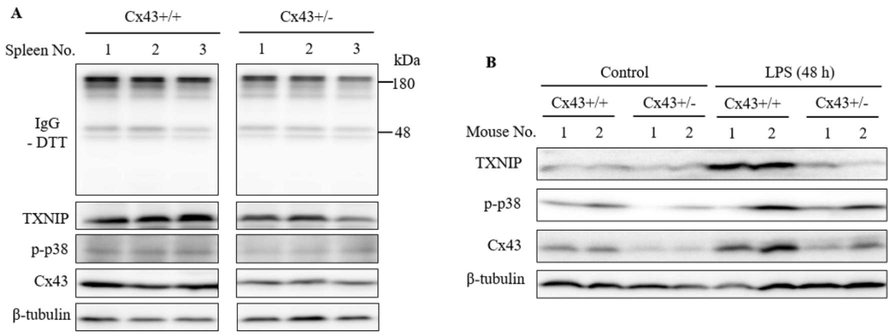

Supplementary Figure S1. (A, B) Levels of IgG and oxidative markers in Cx43$^{+/+}$ and Cx43$^{+/-}$ spleen tissues under basal and LPS-stimulated condition. Spleens from normal Cx43$^{+/+}$ or Cx43$^{+/-}$ mice or mice after administration of 30 mg/kg LPS for 48 h were collected and assayed for level of IgG, TXNIP, p-p38 and Cx43 using Western blot analysis. Note the difference in spleen IgG, TXNIP and Cx43 between Cx43$^{+/+}$ and Cx43$^{+/-}$ mouse under both basal and LPS-stimulated conditions.
